# The Efficacy of Hispidin and Magnesium Nanoparticles against Zearalenone-Induced Fungal Toxicity Causing Polycystic Ovarian Syndrome in Rats

**DOI:** 10.3390/biomedicines12050943

**Published:** 2024-04-24

**Authors:** Amenah Alenazi, Promy Virk, Reem Almoqhem, Amani Alsharidah, Muath Q. Al-Ghadi, Waleed Aljabr, Fawaz Alasmari, Gadah Albasher

**Affiliations:** 1 Department of Zoology, College of Science, King Saud University, Riyadh 11459, Saudi Arabia; 442203638@student.ksu.edu.sa (A.A.); bvirk@ksu.edu.sa (P.V.); ralmoghem@ksu.edu.sa (R.A.); 441203711@student.ksu.edu.sa (A.A.); malghadi@ksu.edu.sa (M.Q.A.-G.); 2 Department of Biological Sciences, College of Science, Northern Border University, Arar 73213, Saudi Arabia; 3 King Fahad Medical City, Riyadh 11525, Saudi Arabia; waljabr@kfmc.med.sa; 4 Department of Pharmacology and Toxicology, College of Pharmacy, King Saud University, Riyadh 11459, Saudi Arabia; ffalasmari@ksu.edu.sa

**Keywords:** zearalenone, hispidin, polycystic ovarian syndrome, aromatase (*Cyp19α1*) gene, mycotoxins, sex hormone-binding globulin (SHBG)

## Abstract

Contamination by fungi and the toxins they secrete is a worldwide health concern. One such toxin is zearalenone (Zea), which is structurally similar to the hormone estrogen, interferes with its action on the reproductive system, and is therefore classified as an endocrine disruptor. This study aims to determine the effectiveness of hispidin and magnesium nanoparticles (MgONPs) against zearalenone-induced myotoxicity, which causes polycystic ovary syndrome (PCOS) in rats. A three-month exposure study was performed using female Wistar rats (*n* = 42) with an average weight of 100–150 g. The animals were divided into six groups (I to VI) of seven rats each. Group I was administered distilled water as a negative control. Group II was exposed to Zea 0.1 mg/kg b.w. through gavage daily. Group III was treated with 0.1 mg/kg of hispidin through gavage daily. Group IV was given 150 µg/mL MgONPs orally each day. Group V was treated with Zea 0.1 mg/kg b.w. + 0.1 mg/kg hispidin orally each day. Group VI was treated with Zea 0.1 mg/kg b.w. and the combination treatment of 0.1 mg/kg hispidin + 150 µg/mL MgONPs through gavage every day. The effectiveness of hispidin and MgONPs against Zea toxicity was evaluated in terms of ovarian histological changes, gene expression, oxidative stress biomarkers, biochemical variables, and hormone levels. The findings showed that exposure to Zea promotes PCOS in rats, with Zea-treated rats displaying hyper-ovulation with large cysts; elevated testosterone, luteinizing hormone, insulin, and glucose; and reduced sex hormone-binding globulin. In addition, qRT-PCR for aromatase (*Cyp19α1*) showed it to be downregulated. Treatment with hispidin improved the histopathological and hormonal situation and rescued expression of *Cyp19α*. Our data indicate the potential therapeutic effects of hispidin against Zea-induced Fungal Toxicity.

## 1. Introduction

Contamination of crops is among the greatest agricultural concerns, particularly contamination by filamentous fungi, which create mycotoxins as secondary metabolites. These mycotoxins can enter the food chain through the consumption of contaminant crops and are able to resist decomposition and being broken down by the digestive systems of animals or humans [1]. Fungal contamination can occur in a number of stages as a result of crop handling and the numerous biological and ecological factors that contribute to fungal growth and mycotoxin production [2]. These mycotoxins pose considerable risk to public health by causing both short-term and long-term symptoms; consequently, several countries, including the Gulf Cooperation Council (GCC), have placed restrictions on feed and food [3]. Importantly, mycotoxins may induce adverse effects even when exposed to a low concentration [4]. 

One of the most notorious fungal genera is *Fusarium*, an old genus that is common in Asia, America, and Europe [5]. In terms of animal wellness and efficiency, three of the most important classes of mycotoxins generated by *Fusarium* species are fumonisins (FBs), zearalenone (Zea), and trichothecenes (deoxynivalenol [DON], nivalenol [NIV], T-2, and HT-2 toxins) [6]. 

Zea is characterized by its high resistance to technological treatments, which makes it difficult to eliminate from human and animal food [7]. Also, Zea has a tremendous economic effect due to it negatively impacting the contaminated crops [8]. Several studies have provided evidence supporting that animal and human exposure to Zea can result in reproductive defects and impair the development of sperm and oocytes [9,10]. Structurally, Zea is an analog of the hormone estrogen and hence is able to disrupt the synthesis and secretion of steroid hormones, interaction with the estrogen receptor, and estrogen-negative feedback regulation, hence its effects on ovulation and spermatogenesis [11]; accordingly, it is described as a hormone disruptor [12]. 

Polycystic ovarian syndrome (PCOS) is a metabolic disorder and multisystem disease that constitutes the main reason for anovulation and failure of female fertility [13]. Scientists have a growing awareness of the features and causes of this disease, with particular interest having developed in the association of PCOS with environmental pollutants such as estrogenic mycotoxins [14]. PCOS is characterized by altered expression of the genes and enzymes responsible for the development of ovulation, including the cytochrome P450 genes, designated with the root symbol *CYP* for human genes [15]. This family includes enzymes involved in active steroid hormone biosynthesis, such as CYP19, which in humans and rats is predominantly expressed in the preovulatory follicle, the corpus luteum of ovulatory humans, and the corpus luteum of rats during the second half of pregnancy [16].

PCOS also has an inverse relationship with sex hormone-binding protein (SHBG), a 93.4-kDa glycated homo-dimeric plasma transport glycoprotein produced by hepatocytes that binds to and regulates the levels of sex hormones in the blood [17]. SHBG is regarded as the primary plasma transporter of testosterone (T), estradiol (E2), and other sex steroids, which it binds to with high affinity [18]. As such, it controls the bioavailability of sex steroid hormones. Notably, in addition to PCOS, SHBG has inverse correlations with obesity, insulin resistance, metabolic syndrome, and type 2 diabetes, all of which has led to increasing interest in this protein [19].

In China, Korea, and other Asian countries, hemorrhage, hemostasis, and conditions associated with female reproductive health are traditionally treated with the medicinal mushroom *Phellinus linteus* (“Sanghuang” in Chinese) [13]. Recently, a large number of bioactive components have been isolated from *P. linteus*, particularly one identified as hispidin, and their biological activities have been confirmed [20]. Hispidin is notably characterized as having strong antioxidant, anticancer, and antidiabetic properties [21]. The present study aimed to determine the effectiveness of hispidin and magnesium oxide nanoparticles (MgONPs) in a rat model of mycotoxin (zearalenone)-induced PCOS.

Thus, the present study aims to assess the efficacy of hispidin and magnesium oxide nanoparticles against zearalenone induced polycystic ovarian syndrome and associated toxicity in female rats. Particularly, our work investigates the protective effects of hispidin and magnesium nanoparticles and their combinations against changes in biochemical parameters, oxidative stress markers, sex hormones, and *Cyp19α1* gene level, as well as histopathological alterations in the ovary in zearalenone-induced PCOS in rats. 

## 2. Materials and Methods

### 2.1. Animals

Forty adults female Wistar albino rats (age 9–10 weeks, 100–150 g) were obtained from the animal house facility at the Department of Zoology, College of Science, King Saud University, Riyadh, Saudi Arabia. All animals were acclimated for one week under controlled environmental conditions (25 ± 1 °C temperature, 50 ± 10% humidity, and 12/12 hr light/dark cycles) before the experimental period. Animals were provided with water and food ad libitum. All experimental protocols were approved by the King Saud University ethics committee (Riyadh, Saudi Arabia; approval no KSU-SE-22-67).

### 2.2. Chemicals

Zea ((HPLC) ≥ 99.0%) and hispidin ((HPLC) ≥ 99.9%) were purchased from Med Chem Express Company, USA. Magnesium oxide nanoparticles (MgONPs) were commercially purchased from Sigma-Aldrich (St. Louis, MO, USA).

### 2.3. Preparation of Zea and Hispidin Solutions

The mycotoxin (zearalenone powder, 10 mg) was first dissolved in 3 mL of ethanol, then added to 997 mL of distilled water, kept in a bottle (1000 mL) and stored at −20 °C. Hispidin powder (10 mg) was likewise dissolved in 3 mL of ethanol, then added to 997 mL distilled water and stored in a 1000 mL bottle at −20 °C. Additional solution was prepared as needed until the end of the exposure period. 

### 2.4. Experimental Design

The study sample consisted of 42 female rats, which were divided randomly into six groups (*n* = 7 in each group). Zea was used to induce PCOS in the experimental groups [22]. Group I: non-treated, considered as a negative control. Group II: received Zea at 0.1 mg/kg b.w. through gavage [22]. Group III: received 0.1 mg/kg of hispidin through gavage. Group IV: received 150 µg/mL MgONPs through gavage [23]. Group V: received Zea at 0.1 mg/kg b.w. + 0.1 mg/kg hispidin through gavage. Group VI: received Zea at 0.1 mg/kg b.w. + 0.1 mg/kg hispidin + 150 µg/mL MgONPs through gavage. All treatments were administered daily for three months (the period of the experiment). 

### 2.5. Sampling and Tissue Preparation

At completion of the study period, whole blood was immediately collected without an anticoagulant, incubated at 37 °C for 30 min, and centrifuged at 3000× *g* for 10 min to obtain serum for the assessment of biochemical parameters. Thereafter, the animals were sacrificed, and the ovaries were extracted for the determination of zearalenone toxicity. A set of ovarian samples was reserved for biochemical assays. All serum and tissue samples were stored at −80 °C until further analysis.

### 2.6. Biochemical Assays

Glucose levels and total cholesterol, triglyceride, high-density lipoprotein (HDL), and low-density lipoprotein (LDL) were evaluated using lab-care diagnostic kits. Commercial ELISA kits (MyBioSource, San Diego, CA, USA) were used to assay serum hormone levels: insulin (MBS724709), LH (MBS700807), testosterone (MBS2563818), and SHBG (MBS902439), all according to the manufacturer’s protocol. 

### 2.7. Determination of Biomarkers of Oxidative Stress

Commercial ELISA kits (MyBioSource, San Diego, CA, USA) were used to determine serum levels of glutathione (GSH; MBS265966) and malondialdehyde (MDA; MBS268427) according to standard ELISA protocols. 

### 2.8. Quantitative Real-Time Polymerase Chain Reaction (qRT-PCR) for Aromatase (Cyp19α1) 

Total RNA was extracted from female rat ovarian tissue samples using the Pure Link RNA Mini Kit (Ambion, Austin, TX, USA) following the manufacturer’s instructions. The quantity and purity of the obtained RNA were measured with a NanoDrop spectrophotometer. Subsequently, cDNA synthesis was carried out using the High-Capacity cDNA Reverse Transcription Kit (Applied Biosystems, Thermo Fisher Scientific, Waltham, MA, USA) and oligo-dT following the manufacturer’s protocol. 

The cDNA samples were then subjected to qPCR using primers specific for *Cyp19α1*: forward 5′-CTGCTGATCATGGGCCTCCT-3′ and reverse 5′-CTCCACAGGCTCGGGTTGTT-3′. The amplification protocol consisted of 40 cycles of denaturation at 95 °C for 45 s, annealing at 59 °C for 45 s, and extension at 72 °C for 45 s. During the first cycle, the 95 °C step was extended to 4 min. Additionally, *β-actin* was amplified in the same reaction to serve as the reference gene. Each measurement was repeated three times. The obtained values were used to calculate the *Cyp19α1*/*β-actin* ratio, which was normalized to the control (calibrator). Thus, the control group was taken as the basis for the calibration and calculation of Ct values. 

### 2.9. Histopathological Analysis

For histopathological evaluation of ovary tissue, samples were first fixed in 10% formalin, trimmed, dehydrated, and embedded in a paraffin block. Sections were then sliced by a microtome and mounted on glass slides for staining with hematoxylin and eosin. Finally, stained sections were examined under a light microscope at a magnification of 40× for total follicular count (follicles and cystic follicles). 

### 2.10. Statistical Analysis

Data were statistically analyzed using the SPSS software (ver.22; SPSS Inc., Chicago, IL, USA). Group differences were evaluated for significance using one-way ANOVA followed by the Duncan multi comparison test. In all analyses, differences were considered significant at a value of *p* ≤ 0.05.

## 3. Results

Exposure to Zea 0.1 mg/kg b.w. in adult rats and treatment with hispidin and magnesium nanoparticles did not cause any mortality during the experimental period.

### 3.1. Hormones and Biochemical Markers

Serum levels of selected hormones and biochemical markers were determined after isolating serum from the blood with kits according to the associated manufacturer protocols.

#### 3.1.1. Hormones 

The evaluated hormones are enumerated below.

##### Free Testosterone

**Figure 1:**
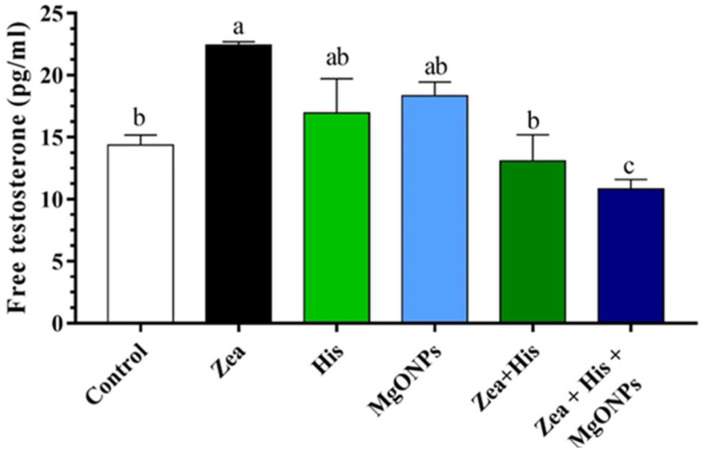
Mean (±SE) serum free testosterone (pg/mL) of rats exposed zearalenone 0.1 mg/kg b.w. (Zea), hispidin (His), or magnesium oxide nanoparticles (MgONPs), and Zea treated with hispidin (Zea + His) or combined hispidin and magnesium oxide nanoparticles (Zea + Hi s+ MgONPs). Different letters indicate significant differences between groups (*p* ≤ 0.05).

Serum free testosterone was significantly (*p* ≤ 0.05) higher in the group exposed to Zea only (22.43 ± 0.27 pg/mL) in comparison to the control (14.36 ± 0.83 pg/mL). Treatment of Zea-exposed rats with hispidin (13.06 ± 2.14 pg/mL) or the hispidin–nanoparticle combination (10.83 ± 0.77 pg/mL) resulted in significantly (*p* ≤ 0.05) reduced free testosterone relative to the group exposed to Zea only. Additionally, free testosterone in treated groups was significantly (*p* ≤ 0.05) higher than in the control group (His, 16.94 ± 2.78 pg/mL; MgONPs, 18.34 ± 1.1 pg/mL). Finally, of the groups exposed to both Zea and a treatment, the rats that received His + MgONPs had significantly (*p* ≤ 0.05) lower serum testosterone than those treated with His alone (Figure 1).

##### Sex Hormone-Binding Globulin (SHBG)

**Figure 2:**
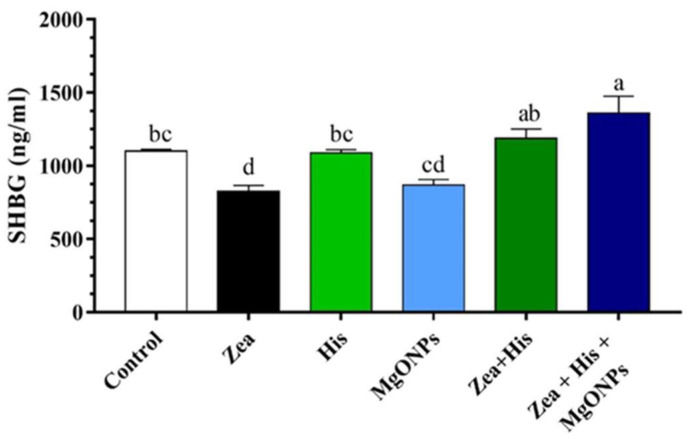
Mean (±SE) serum SHBG (ng/mL) of rats exposed zearalenone 0.1 mg/kg b.w. (Zea), hispidin (His), or magnesium oxide nanoparticles (MgONPs), and Zea treated with hispidin (Zea + His) or combined hispidin and magnesium oxide nanoparticles (Zea + His + MgONPs). Different letters indicate significant differences between groups (*p* ≤ 0.05).

SHBG levels in serum were significantly (*p* ≤ 0.05) reduced in the group exposed to Zea (825.97 ± 39.85 ng/mL) relative to the control (1100.03 ± 13.2 ng/mL). Treatment of Zea-exposed rats with hispidin (1187.25 ± 63.68 ng/mL) or the hispidin–nanoparticle combination (1360.3 ± 116.21 ng/mL) resulted in significantly (*p* ≤ 0.05) higher SHBG levels in comparison to both the Zea-only group and control. SHBG levels in rats exposed to hispidin alone (1087.71 ± 23.01 ng/mL) were not significantly different from those in the control group; meanwhile, levels in rats exposed to MgONPs alone (869.24 ± 36.34 ng/mL) were significantly (*p* ≤ 0.05) reduced from the control group, but higher than in rats exposed to Zea. Finally, rats receiving the His + MgONPs combination treatment exhibited significantly (*p* ≤ 0.05) higher serum SHBG compared to the group treated with His only (Figure 2).

##### Luteinizing Hormone (LH) 

**Figure 3:**
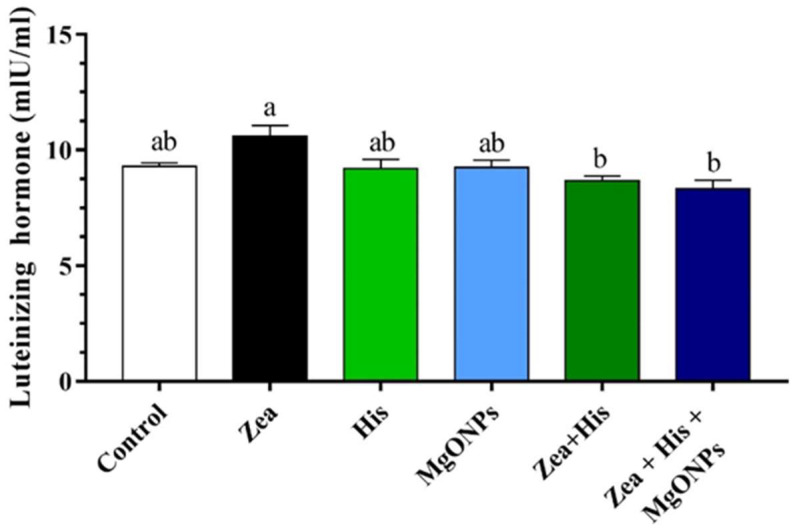
Mean (±SE) serum lutenizing hormone (LH, mlU/mL) of rats exposed zearalenone 0.1 mg/kg b.w. (Zea), hispidin (His), or magnesium oxide nanoparticles (MgONPs), and Zea treated with hispidin (Zea + His) or combined hispidin and magnesium oxide nanoparticles (Zea + His + MgONPs). Different letters indicate significant differences between groups (*p* ≤ 0.05).

Serum LH was not found to differ significantly between control rats (9.29 ± 0.16 mlU/mL) and those treated with either His alone (9.2 ± 0.4 mlU/mL) or MgONPs alone (9.24 ± 0.33 mlU/mL). However, LH levels were significantly (*p* ≤ 0.05) higher in the group exposed to Zea only (10.59 ± 0.46 mlU/mL). In rats receiving Zea, treatment with hispidin (8.69 ± 0.19 mlU/mL) or the combination treatment (8.31 ± 0.38 mlU/mL) resulted in significantly (*p* ≤ 0.05) reduced LH relative to those exposed to Zea only. Finally, the group treated with Zea + His and those receiving Zea + His + MgONPs did not differ significantly in terms of serum LH (Figure 3).

##### Insulin 

**Figure 4:**
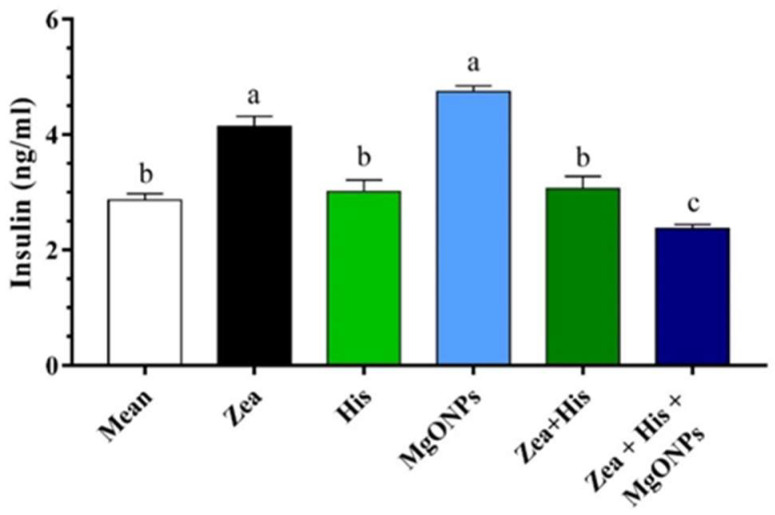
Mean (±SE) serum insulin (ng/mL) of rats exposed zearalenone 0.1 mg/kg b.w. (Zea), hispidin (His), or magnesium oxide nanoparticles (MgONPs), and Zea treated with hispidin (Zea + His) or combined hispidin and magnesium oxide nanoparticles (Zea + His + MgONPs). Different letters indicate significant differences between groups (*p* ≤ 0.05).

With regard to serum insulin, rats receiving Zea alone (4.14 ± 0.18 ng/mL) had significantly higher levels than both control (2.87 ± 0.11 ng/mL) and His-treated rats (3.01 ± 0.2 ng/mL). Meanwhile, exposure to MgONPs alone (4.74 ± 0.1 ng/mL) resulted in significantly (*p* < 0.05) higher insulin levels. In rats that received Zea, treatment with His (3.06 ± 0.22 ng/mL) returned insulin levels to normal, while the His + MgONPs combination treatment (2.37 ± 0.07 ng/mL) not only restored insulin levels but reduced them to below the level of the control group. Accordingly, the rats that received the combination treatment (Zea + His + MgONPs) had insulin levels significantly lower than those that received Zea + His (Figure 4). 

#### 3.1.2. Biochemical Markers

##### Glucose

**Figure 5:**
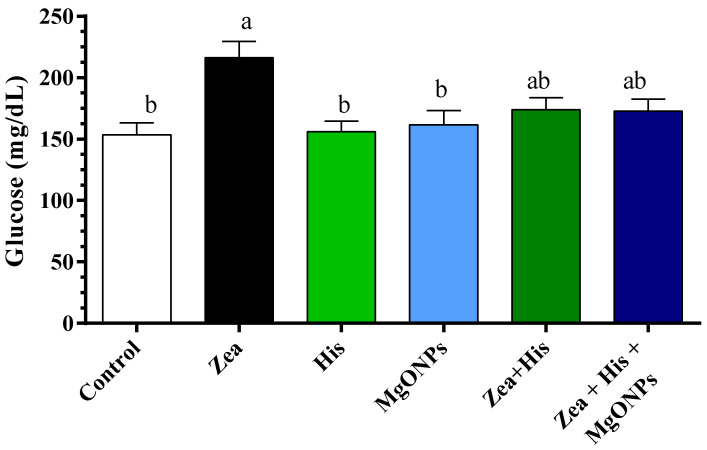
Mean (±SE) serum glucose (mg/dL) of rats exposed zearalenone 0.1 mg/kg b.w. (Zea), hispidin (His), or magnesium oxide nanoparticles (MgONPs), and Zea treated with hispidin (Zea + His) or combined hispidin and magnesium oxide nanoparticles (Zea + His + MgONPs). Different letters indicate significant differences between groups (*p* ≤ 0.05).

Glucose levels did differ significantly (*p* ≤ 0.05) among groups, with higher levels in the group exposed to Zea only (216.3 ± 13.23 mg/dL) compared to the control (153.4 ± 9.76 mg/dL), rats treated with His alone (156.04 ± 8.72 mg/dL), and those treated with MgONPs alone (161.64 ± 11.5 mg/dL). Rats receiving MgONPs only or hispidin did not differ significantly from controls. The Zea-induced increase in serum glucose was significantly (*p* ≤ 0.05) reduced by treatment with either hispidin (173.88 ± 9.75 mg/dL) or the combination treatment (His + MgONPs, 172.79 ± 9.73 mg/dL). No difference was observed between the groups treated with Zea + His and with Zea + His + MgONPs (Figure 5).

##### Lipid Profiles

**Figure 6:**
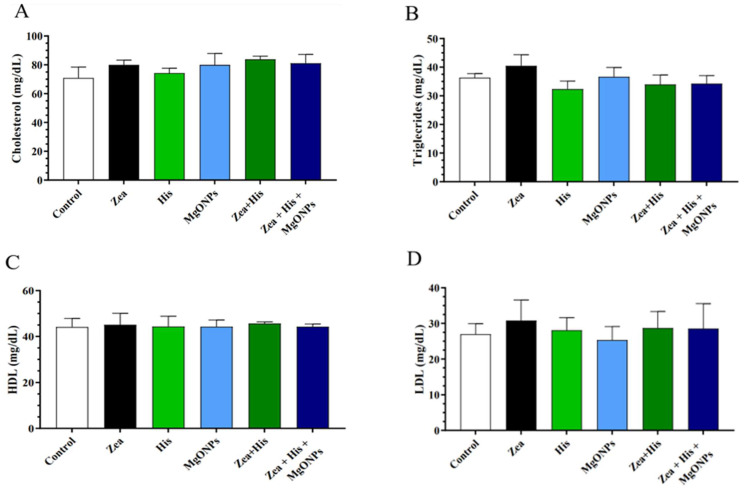
Mean (±SE) serum lipid levels: (**A**) cholesterol, (**B**) triglycerides, (**C**) HDL, (**D**) LDL (mg/dL) of rats exposed to zearalenone 0.1 mg/kg b.w. (Zea), hispidin (His), and/or magnesium oxide nanoparticles (MgONPs) and Zea treated with hispidin (Zea + His) or combined hispidin and magnesium oxide nanoparticles (Zea + His + MgONPs).

Lipid profiles in the groups receiving single treatments did not differ significantly from each other or the controls. Additionally, the two Zea-plus-treatment groups showed no significant difference from the Zea-only group (Figure 6).

### 3.2. Oxidative and Antioxidative Biomarkers

#### 3.2.1. Lipid Peroxides (Malonaldehyde, MDA)

**Figure 7:**
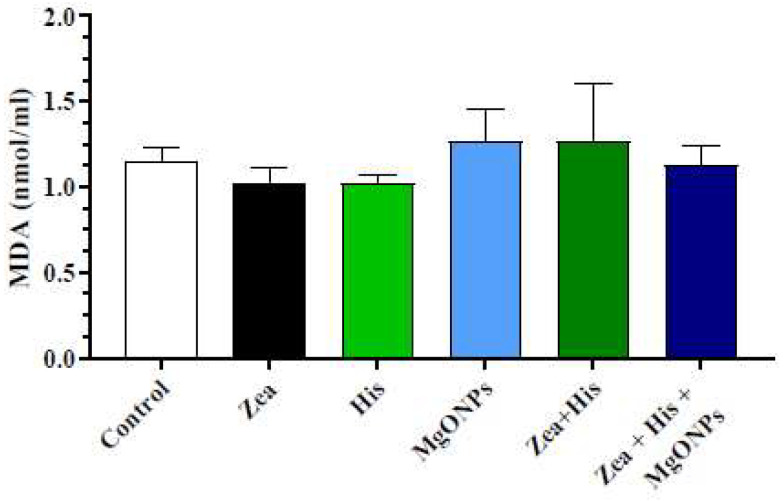
Mean (±SE) serum MDA levels (nmol/mL) of rats exposed to zearalenone 0.1 mg/kg b.w. (Zea), hispidin (His), and/or magnesium oxide nanoparticles (MgONPs) and Zea treated with hispidin (Zea + His) or combined hispidin and magnesium oxide nanoparticles (Zea + His + MgONPs).

Serum MDA levels were found not to significantly (*p* ≤ 0.05) differ in the Zea-exposed group (1.03 ± 0.09) compared to the control (1.15 ± 0.08), His-treated (1.02 ± 0.05), and MgONPs-treated (1.27 ± 0.18) groups. Likewise, concomitant treatment with Zea and hispidin (1.26 ± 0.34) or the combination (Zea + His + MGONPs) (1.13 ± 0.12) did not result in a significant difference compared to either the control group or those exposed to Zea only (Figure 7).

#### 3.2.2. Glutathione (GSH)

**Figure 8:**
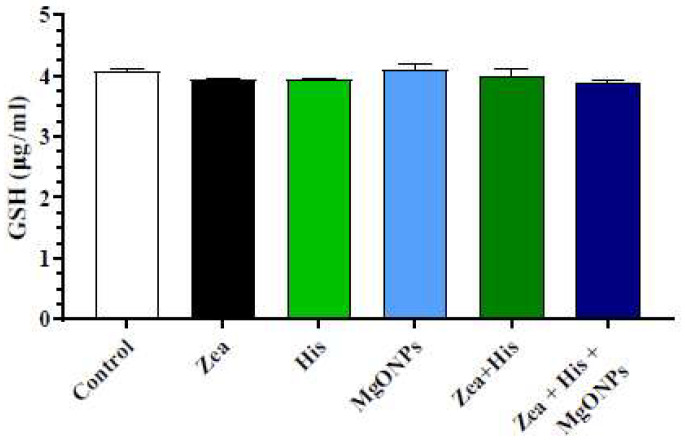
Mean (±SE) serum GSH levels (μg/mL) of rats exposed to zearalenone 0.1 mg/kg b.w. (Zea), hispidin (His), and/or magnesium oxide nanoparticles (MgONPs) and Zea treated with hispidin (Zea + His) or combined hispidin and magnesium oxide nanoparticles (Zea + His + MgONPs).

Serum GSH levels did not differ significantly between rats exposed to Zea and the other single-treatment or control groups. Likewise, treatment of Zea-exposed rats with hispidin or His + MgONPs did not result in a significant difference from other groups (Figure 8).

### 3.3. Aromatase (Cyp19α1) Gene Expression in Ovaries 

**Figure 9:**
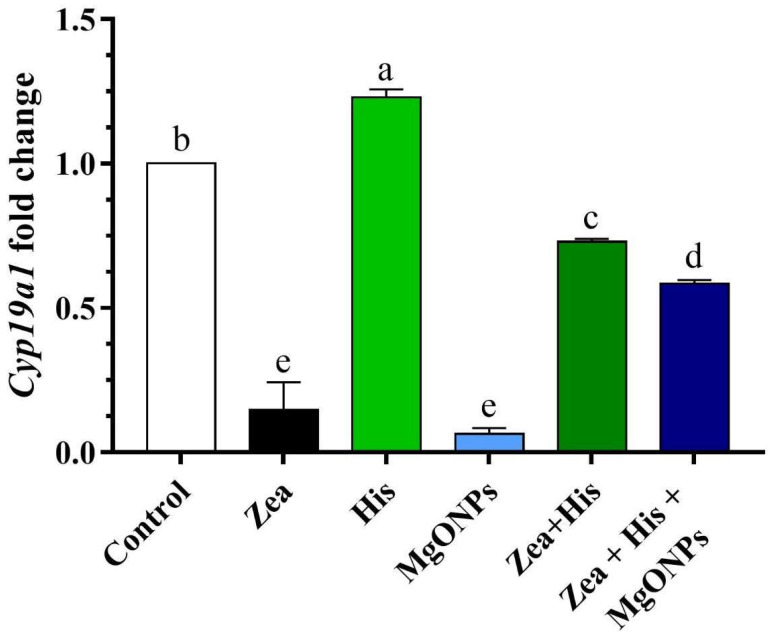
Mean (±SE) expression level of *Cyp19α1* in rats exposed to zearalenone 0.1 mg/kg b.w. (Zea), hispidin (His), and/or magnesium oxide nanoparticles (MgONPs) and Zea treated with hispidin (Zea + His) or combined hispidin and magnesium oxide nanoparticles (Zea + His + MgONPs). Different letters indicate significant differences between groups (*p* ≤ 0.05).

Quantitative real-time PCR was employed to evaluate mRNA expression of aromatase, a key enzyme in the steroid biosynthesis pathway. This revealed significant downregulation in the Zea-exposed group (0.15 ± 0.10 ^e^) and in the MgONPs-treated group (0.06 ± 0.02 ^e^) compared to the negative control (1.0 ± 0.0 ^b^). In rats exposed to Zea, treatment with either hispidin (0.73 ± 0.01 ^c^) or the combination of His + MgONPs (0.58 ± 0.01 ^d^) significantly (*p* ≤ 0.05) upregulated aromatase expression compared to the untreated Zea group. Finally, the group exposed to hispidin only (1.23 ± 0.03 ^a^) exhibited a significantly higher (*p* ≤ 0.05) aromatase level compared to the control group (1.0 ± 0.0 ^b^) (Figure 9).

### 3.4. Ovarian Histopathology

**Figure 10:**
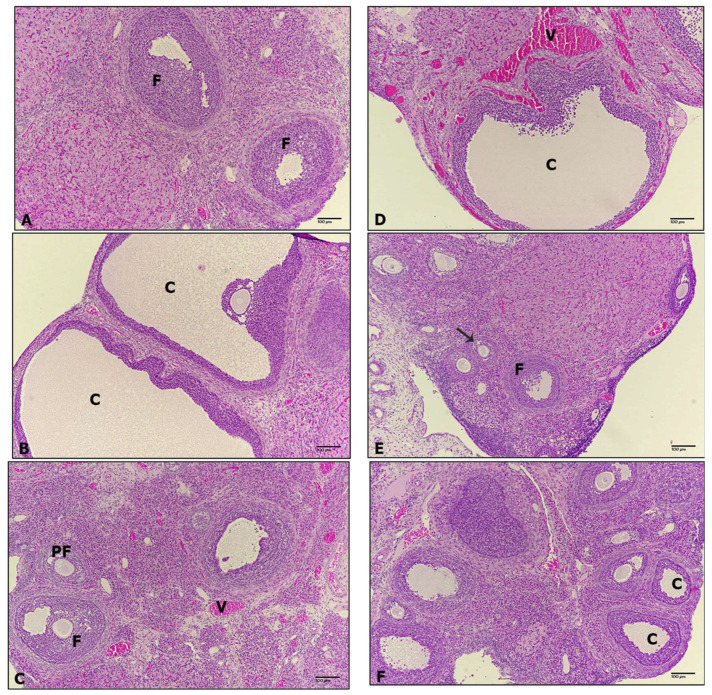
Representative images of histological sections from rat ovaries in this study: (**A**) Control, (**B**) Zea, (**C**) His, (**D**) MgONPs, (**E**) Zea + His, and (**F**) Zea + His + MgONPs. Magnification: 100×; Staining: H & E. C, Cysts; F, Follicles; PF, Primary Follicle; V, Blood Vessel.

Figure 10 shows representative histological sections of rat ovaries. Control rats showed normal structure with various stages of oogenesis (Figure 10A). Rats treated with Zea (0.1 mg/kg b.w.) displayed hyper-ovulation with large cysts (Figure 10B). Meanwhile, those treated with His alone (0.1 mg/kg b.w.) showed a healthy ovarian structure with various stages of oogenesis and no cysts (Figure 10C), but rats treated with MgONPs (150 µg/mL) exhibited cysts and dilatation of blood vessels (Figure 10D). Rats that received both Zea and His demonstrated a marked improvement, in that no cysts were evident (Figure 10E). Finally, rats treated with Zea and combined His and MgONPs displayed some hyper-ovulation with small cysts (Figure 10F).

## 4. Discussion

Hormonal balance is important to good reproductive function, and any disturbance in this balance can lead to adverse effects in the reproductive system and related systems. PCOS is characterized by striking changes in levels of LH, FSH, testosterone, and other hormones related to steroidogenesis and folliculogenesis; in particular, hyperandrogenism is a major characteristic of women with PCOS symptoms [24]. PCOS causes the pituitary to secrete high levels of LH, and the ovaries to produce high levels of androgens. Notably, as a structural analog of estrogen, Zea and its metabolites exert estrogen-like effects and can engage in estrogen-negative feedback regulation to affect hormone biosynthesis, including production of FSH and LH [11].

In the current study, rats exposed to Zea had substantially higher serum free testosterone than the control group. Several investigations corroborate that conclusion, including a prior in vivo study [25] that clearly demonstrated exposure to Zea to delay Leydig cell regeneration and lower androgen production, possibly via altering expression of genes important to Leydig cell development and steroidogenesis in males. Zea has likewise been shown to decrease sperm number and motility along with plasma testosterone levels [26], and such a decrease in testosterone is considered to indicate that Zea damages the testicular tissue from which testosterone is produced [27]. 

The present study also found the rats that received Zea to have significantly higher serum LH than the control group. In addition, the groups exposed to Zea and to MgONPs displayed high serum insulin concentrations, and the group exposed to Zea further exhibited significantly elevated serum glucose. Meanwhile, rats exposed to Zea alone had considerably lower serum SHBG. Overall, the present results are in agreement with prior findings [22] of increased LH, insulin, glucose, and testosterone in rats treated with Zea. Notably, one arm of PCOS pathogenesis is the somatotrophic axis, which involves growth hormone (GH) and insulin-like growth factors (IGFs). Insulin resistance and hyperinsulinemia are common features of patients who have PCOS [28]. The increased IGF-1 activity works to organize ovarian follicular maturation, steroidogenesis, and ovarian hyperandrogenism in PCO individuals [29], while GH helps stimulate β-cell proliferation, which leads to the production and secretion of insulin [30]. 

In PCOS, the ovary may cause a local inflammatory response that stimulates ovarian androgen production [31]; an in vitro study has demonstrated anti-inflammatory agents such as resveratrol to inhibit the ovarian steroidogenic enzyme induced by proinflammatory stimuli [32]. Notably, pro-inflammatory cytokines such as IL-6 and TNF-α play important roles in the pathogenesis of many diseases [33], and TNF-α is capable of stimulating the in vitro proliferation of androgen-producing theca cells [22,34] showed administration of Zea to female rats for three months to increase plasma TNF-α and the expression of Secreted frizzled-related protein 4 (SFRP4), an adipokine involved in the apoptotic process during ovulation and energy metabolism; therefore, this is a deterministic factor in the progress of PCOS.

The liver manufactures SHBG, which as a sex hormone transporter can be utilized to gauge hyperandrogenism. That is, SHBG binds with high affinity to circulating sex steroids, alters the amount of biologically active sex hormones in the blood, and thereby influences how bioavailable they are [35]. Insulin and androgens restrict the liver from producing and releasing SHBG; thus, hyperandrogenism in people with PCOS results in low serum SHBG, which further increases testosterone levels [36]. The hypothalamic–pituitary–ovarian axis is influenced by the amount of androgen in the blood; decreased SHBG synthesis increases the bioavailability of androgen, which in turn causes abnormality in ovarian function [37]. Genetic polymorphisms in *SHBG* can also contribute to the etiology of PCOS [38]. 

Several studies have mentioned overweight or obese women with PCOS to have reduced serum Mg [39], which makes Mg supplementation of interest in this disease. The present study found that MgONP treatment does not affect LH and glucose levels compared with the control but does increase testosterone and insulin. Meanwhile, in Zea-exposed rats, combining MgONPs with hispidin decreases the levels of testosterone, insulin, and glucose compared with the untreated exposed group. This largely agrees with a prior report [40] that markers of inflammation, oxidative stress, or metabolism in PCOS are not significantly altered when magnesium supplementation is administered alone. In a trial to detect the influence of MgO in patients with PCOS, no significant effect was observed after eight weeks of intervention [41]. The present study is the first to investigate the effect of MgONPs in rat models of PCOS, while the MgO appears to have no effect on PCOS. 

All groups in the present study showed no significant differences in lipid profile (cholesterol, triglycerides, HDL, LDL). These findings are in relative alignment with a recent study [42] reporting that a single intraperitoneal dose of zearalenone (2 mg/kg b.w.) on each of three weeks showed no significant induction of HDL and LDL but did increase cholesterol and triglycerides. Meanwhile, another study [43] reported oral administration of Zea (40 μg/kg b.w.) for three weeks to induce significant elevation in triglycerides, cholesterol, and LDL and concomitant significant reduction in HDL. These discrepant and conflicting results may be attributable to differences in experimental design and sample size, and furthermore may depend on Zea dose and duration. In the same line, treatment of Zea-exposed rats with the polyphenol resveratrol was not found to have any significant effect on total cholesterol, LDL, and HDL [42].

The present study also observed all control and treatment groups to have no significant difference in serum MDA and GSH levels. Thus, treatment with 0.1 mg/kg b.w. zearalenone in female rats does not appear to impact oxidative and antioxidative biomarkers. However, several studies have indicated high concentrations of Zea to have an oxidative stress effect: in piglets with concentrations of 1–3 mg/kg b.w. [44], in female rats treated with 50–150 mg/kg b.w. on gestation days 0 through 7 [45] and in female mice given 20–30 mg/kg b.w. [46,47]. Likewise, an in vitro study using porcine IPEC-J2 cells showed that only the highest concentration of Zea tested increased levels of MDA and decreased levels of antioxidants [48]. 

In the same vein, the current study determined that treatment with MgONPs at a concentration of 150 µg/mL did not affect serum levels of MDA and GSH. This finding is in alignment with [23], who also reported that treatment of rats with low concentrations of MgO nanoparticles did not induce any apparent toxicity and concluded that concentrations lower than 250 µg. mL/b.w. were safe for desired applications. Also, ref. [49] found that MgONPs at a dose of 250 mg/kg resulted in no significant change of MDA and GSH in the liver, but doses of 500 mg/kg and higher produced significant effects in a dose-dependent and gender-independent manner.

Interestingly, in the present study, treatment of Zea-exposed rats with hispidin and the combination of hispidin with MgONPs clearly reduced free testosterone and insulin levels and increased serum SHBG relative to the untreated toxin-exposed group. The exact mechanisms by which hispidin exerts its protective effects against Zea-induced reproductive toxicity remain to be elucidated, but it may be that hispidin protects steroidogenesis genes from DNA methylation and associated silencing of transcription.

Aromatase (*CYP19α1*) is a steroidogenic enzyme that catalyzes the process of androgen aromatization in granulosa cells, which is responsible for the conversion of testosterone to estradiol and estrone [50] Indeed, gene expression of *Cyp19α1* is an important marker in PCOS determination [51]. The current study showed downregulation of *Cyp19α1* mRNA in the Zea and MgONPs groups compared with the control group. Several prior studies support this gene and its expression level as being related to PCOS [52,53,54,55]. Importantly, the present study showed treatment with hispidin to result in upregulation of *Cyp19a1* in ovarian tissue due to its capacity to regulate hormones and suppress their impact on aromatase (*CYP19*). In the same line, resveratrol has been demonstrated to be effective against Zea-induced cytotoxicity [42,56]. 

Methylation modification of DNA is a mechanism by which environmental factors can affect gene expression without altering the genetic code [57]. Hypermethylation of *Cyp19α1* has been established to play a major role in promoting PCOS through suppressing transcription of the gene [58]. Nanoparticles (NPs) have been demonstrated to impact reproduction through affecting DNA methylation patterns in the germ line. Importantly, NPs are able to breach the blood–testis, placental, and epithelial barriers, which collectively protect reproductive tissues, on account of their tiny size and other characteristics. NPs then build up in reproductive tissues, such as the ovary, where they cause dysfunction that negatively impacts morphology, decreases the quantity of mature oocytes, and impairs the growth of follicles [59]. The effect of NPs on DNA methylation might be direct or indirect through NP-induced alterations in sex hormones [60]. 

In the present study, administration of Zea and MgONPs alike was found to downregulate *Cyp19a1* in ovarian tissue, which caused hyperandrogenism. Hyperandrogenism then prevents the negative feedback normally provided by LH: increased androgen or low levels of estrogen eventually leads to hypersecretion of LH, which then interacts with the LH receptor in theca cells [54]. In PCOS, increased LH secretion stimulates theca cells to synthesize testosterone, but the concomitant decrease in aromatase activity leads to disorder in the aromatization process: granulosa cells are unable to aromatize androgen into estrogen, there is insufficient estrogen for oocyte maturation, and therefore chronic anovulation results, which is the major feature of PCOS [53].

In the ovary, Zea increases granulosa cell apoptosis in a dose-dependent manner, thereby disrupting follicular development [61]. Azouz, et al. [62] reported elevated expression of the cellular proliferation indicator Ki-67 in theca cell layers in response to excess androgen produced by interstitial cells in the PCOS group, indicating that ovarian cysts are formed through apoptosis of both ovarian oocytes and granulosa cells. In addition to Zea, other compounds that have long been released into the environment can mimic the action of endogenous estrogen, possibly disrupting endocrine function in mammals; these toxicants can also interfere with ovarian function either directly or indirectly through effects on the hypothalamus and pituitary gland [22,63].

Histopathology of ovary tissues in the present work showed that the rats treated with Zea 0.1 mg/kg b.w. for three months to have significant hyper-ovulation with large cysts (visualized by H&E staining). This is in line with previous studies that also used in vivo rat models, which reported predominant changes in the structures of ovarian follicles and provided evidence linking chronic low-dose Zea exposure with PCOS symptom progression [22]. Another study that examined the toxicity of Zea on reproductive organs in mice, in which 2.5 mg/kg of Zea was delivered by intraperitoneal injection on alternate days over a 30-day period, reported decreased uterus size and abnormal characteristics in ovaries; extending the treatment period to 60 and 90 days resulted in additional detrimental effects on ovarian histology [64].

The present study also showed that treatment of rats with magnesium oxide nanoparticles produced ovarian cysts. MgONPs may induce DNA damage in the ovary. In a toxicokinetic analysis, [65] showed that magnesium accumulated significantly in the liver and kidney and altered those tissues. A recent study on fish also reported that exposure to MgONPs resulted in hematologic and histopathological changes [66]. In a subsequent study, [49] orally administered various doses of MgONPs (250, 500, and 1000 mg/kg) to Wistar rats over 28 days and observed a significant induction of DNA damage and chromosomal aberrations. In the same vein, several studies mentioned adverse histopathologic effects of oxide NPs; in particular, Fe_2_O_3_ and NiO NPs produce hepatic and kidney tissue alterations in rats [67,68]. Notably, the present study also observed greater induction of aromatase expression in Zea-exposed rats when MgONPs were administered in combination with hispidin. This work is the first report of MgONP toxicity on the reproductive system; more studies remain needed to fully characterize their effects and elucidate the underlying mechanisms. 

Meanwhile, rats treated with hispidin alone in the current study exhibited a healthy ovarian structure with various stages of oogenesis and no cysts. Interestingly, Zea-exposed rats treated with hispidin demonstrated a marked improvement relative to the untreated exposed group, in that no cysts were seen. These results further support that hispidin may rescue the negative effects of Zea on the female reproductive system and suggest its therapeutic utility in protecting granulosa cells from apoptosis. The anti-apoptotic properties of hispidin have been previously demonstrated in H9c2 cardiomyoblast cells exposed to H2O2 [69] in which hispidin treatment improved the viability of peroxide-exposed cardiomyoblast cells, protecting them against oxidative stress by regulating apoptosis-related proteins such as caspase-3, Bax, and Bcl-2 and by activating the Akt/GSK-3β and ERK1/2 signaling pathways. Additionally, hispidin has demonstrated a protective effect in neurons, preventing mitochondrial dysfunction and cellular apoptosis [70]. 

The topic of polycystic ovary syndrome is broad, as it is a syndrome that is affected by many elements. Therefore, we did not investigate the signaling pathways that might be involved in the pathology of PCOS induced by fungal Zea. Although we investigated the role of the *Cyp19a1* gene level in Zea-induced PCOS, other genes might also be involved in this toxicity effect. The present study did not determine the role of immune inflammatory and apoptosis systems, as they might be associated with this syndrome. Studies are warranted to determine the levels and extents of fungal Zea in the cells.

## 5. Conclusions

The current study showed Zea exposure in rats to cause alterations in serum hormones and gene expression and to lead to hyper-ovulation with large cysts. It also demonstrated hispidin to be safe and efficacious in rescuing the toxicity induced by Zea on the ovary. This protective effect could be attributed to the ability of phenolic compounds to regulate inflammatory and apoptotic activities and prevent DNA damage. Further study is needed to fully characterize the beneficial effects of hispidin and elucidate the underlying mechanisms. More research is recommended to elucidate the role of signaling pathways, including inflammatory, apoptosis and immunology pathways, associated with toxicity of Zea in ovaries functions. A genetic study should be conducted in the near future to highlight the potential genes targeted by hispidin for attenuating PCOS. The present study focused on the molecular changes in the ovarian tissue and serum; however, further studies are recommended to further explore these changes at other locations such as the ovarian hypothalamic axis.

## Data Availability

Data are contained within the article.

## References

[1] Kang’Ethe E.K., A Lang’A K. (2009). Aflatoxin B1 and M1 contamination of animal feeds and milk from urban centers in Kenya. Afr. Health Sci..

[2] Marin S., Ramos A.J., Cano-Sancho G., Sanchis V. (2013). Mycotoxins: Occurrence, toxicology, and exposure assessment. Food Chem. Toxicol..

[3] Al-Jaal B., Salama S., Al-Qasmi N., Jaganjac M. (2019). Mycotoxin contamination of food and feed in the Gulf Cooperation Council countries and its detection. Toxicon.

[4] Tittlemier S.A., Cramer B., Dall’Asta C., Iha M.H., Lattanzio V.M.T., Maragos C., Solfrizzo M., Stranska M., Stroka J., Sumarah M. (2020). Developments in mycotoxin analysis: An update for 2018-19. World Mycotoxin J..

[5] Tiemann U., Brü K.-P., Jonas L., Pö R., Schneider F., Dä S. (2006). Effects of diets with cereal grains contaminated by graded levels of two Fusarium toxins on selected immunological and histological measurements in the spleen of gilts 1,2. J. Anim. Sci..

[6] Summerell B.A., Leslie J.F. (2011). Fifty years of Fusarium: How could nine species have ever been enough?. Fungal Divers..

[7] Boeira S.P., Filho C.B., Del’Fabbro L., Roman S.S., Royes L.F.F., Fighera M.R., Jessé C.R., Oliveira M.S., Furian A.F. (2014). Lycopene treatment prevents hematological, reproductive and histopathological damage induced by acute zearalenone administration in male Swiss mice. Exp. Toxicol. Pathol..

[8] Reddy K.E., Song J., Lee H.J., Kim M., Kim D.W., Jung H.J., Kim B., Lee Y., Yu D., Kim D.W. (2018). Effects of high levels of deoxynivalenol and zearalenone on growth performance, and hematological and immunological parameters in pigs. Toxins.

[9] Yang Z., Xue K.S., Sun X., Williams P.L., Wang J.S., Tang L. (2018). Toxicogenomic responses to zearalenone in Caenorhabditis elegans reveal possible molecular mechanisms of reproductive toxicity. Food Chem. Toxicol..

[10] Yousef M.S., Takagi M., Talukder A.K., Marey M.A., Kowsar R., Abdel-Razek A.R.K., Shimizu T., Fink-Gremmels J., Miyamoto A. (2017). Zearalenone (ZEN) disrupts the anti-inflammatory response of bovine oviductal epithelial cells to sperm in vitro. Reprod. Toxicol..

[11] Zheng W., Feng N., Wang Y., Noll L., Xu S., Liu X., Lu N., Zou H., Gu J., Yuan Y. (2019). Effects of zearalenone and its derivatives on the synthesis and secretion of mammalian sex steroid hormones: A review. Food Chem. Toxicol..

[12] Kowalska K., Habrowska-Górczyńska D.E., Piastowska-Ciesielska A.W. (2016). Zearalenone as an endocrine disruptor in humans. Environ. Toxicol. Pharmacol..

[13] Li Y., Chen C., Ma Y., Xiao J., Luo G., Li Y., Wu D. (2019). Multi-system reproductive metabolic disorder: Significance for the pathogenesis and therapy of polycystic ovary syndrome (PCOS). Life Sci..

[14] Ghanati K., Mahdi J., Shakoori A., Saeed A.-B., Ali K.-R., Parisa S. (2023). The association between polycystic ovary syndrome and environmental pollutants based on animal and human study; a systematic review. Rev. Environ. Health.

[15] Nelson D.R., Koymans L., Kamataki T., Stegeman J.J., Feyereisen R., Waxman D.J., Waterman M.R., Gotoh O., Coon M.J., Estabrook R.W. (1996). P450 superfamily: Update on new sequences, gene mapping, accession numbers and nomenclature. Pharmacogenetics.

[16] Payne A.H., Hales D.B. (2004). Overview of steroidogenic enzymes in the pathway from cholesterol to active steroid hormones. Endocr. Rev..

[17] Kahn S.M., Hryb D.J., Nakhla A.M., Romas N.A., Rosner W. (2002). Beyond Carrier Proteins Sex hormone-binding globulin is synthesized in target cells The sex hormone-binding globulin (SHBG) gene. J. Endocrinol..

[18] Goldman A.L., Bhasin S., Wu F.C.W., Krishna M., Matsumoto A.M., Jasuja R. (2017). A reappraisal of testosterone’s binding in circulation: Physiological and clinical implications. Endocr. Rev..

[19] Deswal R., Yadav A., Dang A.S. (2018). Sex hormone binding globulin—An important biomarker for predicting PCOS risk: A systematic review and meta-analysis. Syst. Biol. Reprod. Med..

[20] Lee I.S., Bae K., Kuk Yoo J., Ryoo I.J., Kim B.Y., Seog Ahn J., Yoo I.D. (2012). Inhibition of human neutrophil elastase by ergosterol derivatives from the mycelium of Phellinus linteus. J. Antibiot..

[21] Chen W., Feng L., Shen Y., Su H., Li Y., Zhuang J., Zhang L., Zheng X. (2013). Myricitrin inhibits acrylamide-mediated cytotoxicity in human caco-2 cells by preventing oxidative stress. BioMed Res. Int..

[22] Abbasian N., Momtaz S., Baeeri M., Navaei-Nigjeh M., Hosseini R., Abdollahi M. (2018). Molecular and biochemical evidence on the role of zearalenone in rat polycystic ovary. Toxicon.

[23] Mazaheri N., Naghsh N., Karimi A., Salavati H. (2019). In vivo toxicity investigation of magnesium oxide nanoparticles in rat for environmental and biomedical applications. Iran. J. Biotechnol..

[24] Rezvanfar M.A., Shojaei Saadi H.A., Gooshe M., Abdolghaffari A.H., Baeeri M., Abdollahi M. (2014). Ovarian aging-like phenotype in the hyperandrogenism-induced murine model of polycystic ovary. Oxidative Med. Cell. Longev..

[25] Zhou S., Wang Y., Ma L., Chen X., Lü Y., Ge F., Chen Y., Chen X., Lian Q., Jin X.D. (2018). Zearalenone delays rat leydig cell regeneration. Toxicol. Sci..

[26] Del Fabbro L., Jesse C.R., de Gomes M.G., Borges Filho C., Donato F., Souza L.C., Goes A.R., Furian A.F., Boeira S.P. (2019). The flavonoid chrysin protects against zearalenone induced reproductive toxicity in male mice. Toxicon.

[27] Long M., Yang S., Zhang W., Zhang Y., Li P., Guo Y., Wang Y., Chen X., He J. (2016). The Influence of Selenium Yeast on Hematological, Biochemical and Reproductive Hormone Level Changes in Kunming Mice Following Acute Exposure to Zearalenone. Biol. Trace Elem. Res..

[28] Rezvanfar M.A., Rezvanfar M.A., Ahmadi A., Shojaei-Saadi H.A., Baeeri M., Abdollahi M. (2012). Molecular mechanisms of a novel selenium-based complementary medicine which confers protection against hyperandrogenism-induced polycystic ovary. Theriogenology.

[29] Erickson G.F., Magoffin D.A., Cragun J.R., Chang R.J. (1990). The Effects of Insulin and Insulin-Like Growth Factors-I and-II on Estradiol Production by Granulosa Cells of Polycystic Ovaries. J. Clin. Endocrinol. Metab..

[30] Fu Z., Gilbert E.R., Liu D. (2013). Regulation of Insulin Synthesis and Secretion and Pancreatic Beta-Cell Dysfunction in Diabetes. Curr. Diabetes Rev..

[31] González F. (2012). Inflammation in Polycystic Ovary Syndrome: Underpinning of insulin resistance and ovarian dysfunction. Steroids.

[32] Ortega I., Villanueva J.A., Wong D.H., Cress A.B., Sokalska A., Stanley S.D., Duleba A.J. (2014). Resveratrol potentiates effects of simvastatin on inhibition of rat ovarian theca-interstitial cells steroidogenesis. J. Ovarian Res..

[33] Turner M.D., Nedjai B., Hurst T., Pennington D.J. (2014). Cytokines and chemokines: At the crossroads of cell signalling and inflammatory disease. Biochim. Et Biophys. Acta.

[34] Spaczynski R.Z., Arici A., Duleba A.J. (1999). Tumor Necrosis Factor-Stimulates Proliferation of Rat Ovarian Theca-Interstitial Cells 1. Biol. Reprod..

[35] Hammond G.L., Wu T.S., Simard M. (2012). Evolving utility of sex hormone-binding globulin measurements in clinical medicine. Curr. Opin. Endocrinol. Diabetes Obes..

[36] Saddick S.Y. (2020). Identifying genes associated with the development of human polycystic ovary syndrome. Saudi J. Biol. Sci..

[37] Chang R.J. (2007). The reproductive phenotype in polycystic ovary syndrome. Nat. Clin. Pract. Endocrinol. Metab..

[38] Xing C., Zhang J., Zhao H., He B. (2022). Effect of Sex Hormone-Binding Globulin on Polycystic Ovary Syndrome: Mechanisms, Manifestations, Genetics, and Treatment. Int. J. Women’s Health.

[39] Babapour M., Mohammadi H., Kazemi M., Hadi A., Rezazadegan M., Askari G. (2021). Associations Between Serum Magnesium Concentrations and Polycystic Ovary Syndrome Status: A Systematic Review and Meta-analysis. Biol. Trace Elem. Res..

[40] Li R., Li Z., Huang Y., Hu K., Ma B., Yang Y. (2022). The effect of magnesium alone or its combination with other supplements on the markers of inflammation, OS and metabolism in women with polycystic ovarian syndrome (PCOS): A systematic review. Front. Endocrinol..

[41] Alizadeh M., Karandish M., Asghari Jafarabadi M., Heidari L., Nikbakht R., Babaahmadi Rezaei H., Mousavi R. (2021). Metabolic and hormonal effects of melatonin and/or magnesium supplementation in women with polycystic ovary syndrome: A randomized, double-blind, placebo-controlled trial. Nutr. Metab..

[42] Virk P., Al-mukhaizeem N.A.R., Bin Morebah S.H., Fouad D., Elobeid M. (2020). Protective effect of resveratrol against toxicity induced by the mycotoxin, zearalenone in a rat model. Food Chem. Toxicol..

[43] El-Nekeety A.A., El-Kady A.A., Abdel-Wahhab K.G., Hassan N.S., Abdel-Wahhab M.A. (2017). Reduction of individual or combined toxicity of fumonisin B1 and zearalenone via dietary inclusion of organo-modified nano-montmorillonite in rats. Environ. Sci. Pollut. Res..

[44] Jiang S.Z., Yang Z.B., Yang W.R., Gao J., Liu F.X., Broomhead J., Chi F. (2011). Effects of purified zearalenone on growth performance, organ size, serum metabolites, and oxidative stress in postweaning gilts. J. Anim. Sci..

[45] Jia Z., Liu M., Qu Z., Zhang Y., Yin S., Shan A. (2014). Toxic effects of zearalenone on oxidative stress, inflammatory cytokines, biochemical and pathological changes induced by this toxin in the kidney of pregnant rats. Environ. Toxicol. Pharmacol..

[46] Liang Z., Ren Z., Gao S., Chen Y., Yang Y., Yang D., Deng J., Zuo Z., Wang Y., Shen L. (2015). Individual and combined effects of deoxynivalenol and zearalenone on mouse kidney. Environ. Toxicol. Pharmacol..

[47] Llorens P., Herrera M., Juan-García A., Payá J.J., Moltó J.C., Ariño A., Juan C. (2022). Biomarkers of Exposure to Zearalenone in In Vivo and In Vitro Studies. Toxins.

[48] Fan W., Shen T., Ding Q., Lv Y., Li L., Huang K., Yan L., Song S. (2017). Zearalenone induces ROS-mediated mitochondrial damage in porcine IPEC-J2 cells. J. Biochem. Mol. Toxicol..

[49] Mangalampalli B., Dumala N., Perumalla Venkata R., Grover P. (2018). Genotoxicity, biochemical, and biodistribution studies of magnesium oxide nano and microparticles in albino wistar rats after 28-day repeated oral exposure. Environ. Toxicol..

[50] Warsy A.S., Almukaynizi F.B., AlDaihan S., Alam S., Daghastani M. (2017). Genetic Polymorphisms in Aromatase (*CYP19*) Gene and Cancer. Genet. Polymorph..

[51] Aghaie F., Khazali H., Hedayati M., Akbarnejad A. (2018). The effects of exercise on expression of *CYP19* and *StAR* mRNA in steroid-induced polycystic ovaries of female rats. Int. J. Fertil. Steril..

[52] Lee Y.H., Yang H., Lee S.R., Kwon S.W., Hong E.J., Lee H.W. (2018). Welsh onion root (*Allium fistulosum*) restores ovarian functions from letrozole induced-polycystic ovary syndrome. Nutrients.

[53] Panghiyangani R., Soeharso P., Pujianto A., Suryandari D., Wiweko B., Kurniati M., Pujianto D. (2020). *CYP19A1* gene expression in patients with polycystic ovarian syndrome. J. Hum. Reprod. Sci..

[54] Suriyakalaa U., Ramachandran R., Doulathunnisa J.A., Aseervatham S.B., Sankarganesh D., Kamalakkannan S., Kadalmani B., Angayarkanni J., Akbarsha M.A., Achiraman S. (2021). Upregulation of Cyp19a1 and PPAR-γ in ovarian steroidogenic pathway by Ficus religiosa: A potential cure for polycystic ovary syndrome. J. Ethnopharmacol..

[55] Yang F., Ruan Y.-C., Yang Y., Wang K., Liang S., Han Y., Teng X.-M., Yang J.-Z. (2015). Follicular hyperandrogenism downregulates aromatase in luteinized granulosa cells in PCOS women. Reproduction..

[56] Sang Y., Li W., Zhang G. (2016). The protective effect of resveratrol against cytotoxicity induced by mycotoxin, zearalenone. Food Funct..

[57] Sharma A., Heuck C.J., Fazzari M.J., Mehta J., Singhal S., Greally J.M., Verma A. (2010). DNA methylation alterations in multiple myeloma as a model for epigenetic changes in cancer. Wiley Interdiscip. Rev. Syst. Biol. Med..

[58] Yu Y.Y., Sun C.X., Liu Y.K., Li Y., Wang L., Zhang W. (2013). Promoter methylation of *CYP19A1* gene in chinese polycystic ovary syndrome patients. Gynecol. Obstet. Investig..

[59] Wang R., Song B., Wu J., Zhang Y., Chen A., Shao L. (2018). Potential adverse effects of nanoparticles on the reproductive system. Int. J. Nanomed..

[60] Anway M.D., Cupp A.S., Uzumcu N., Skinner M.K. (2005). Toxicology: Epigenetic transgenerational actions of endocrine disruptors and male fertility. Science.

[61] Li N., Liu X.L., Zhang F.L., Tian Y., Zhu M., Meng L.Y., Dyce P.W., Shen W., Li L. (2020). Whole-transcriptome analysis of the toxic effects of zearalenone exposure on ceRNA networks in porcine granulosa cells. Environ. Pollut..

[62] Azouz A.A., Ali S.E., Abd-Elsalam R.M., Emam S.R., Galal M.K., Elmosalamy S.H., Alsherbiny M.A., Hassan B.B., Li C.G., El Badawy S.A. (2021). Modulation of steroidogenesis by Actaea racemosa and vitamin C combination, in letrozole induced polycystic ovarian syndrome rat model: Promising activity without the risk of hepatic adverse effect. Chin. Med..

[63] Güney M., Demirin H., Oral B., Özgüner M., Bayhan G., Altuntas I. (2007). Ovarian toxicity in rats caused by methidathion and ameliorating effect of vitamins E and C. Hum. Exp. Toxicol..

[64] Ahmad B., Shrivastava V.K., Saleh R., Henkel R., Agarwal A. (2018). Protective effects of saffron against zearalenoneinduced alterations in reproductive hormones in female mice (Mus musculus). Clin. Exp. Reprod. Med..

[65] Mangalampalli B., Dumala N., Grover P. (2017). Acute oral toxicity study of magnesium oxide nanoparticles and microparticles in female albino Wistar rats. Regul. Toxicol. Pharmacol..

[66] Sudhabose S., Sooryakanth B., Rajan M.R. (2023). Acute Toxicity, Hematological Profile, and Histopathological Effects of MgO Nanoparticles on Gills, Muscle, Liver of Mrigal, Cirrhinus mrigala. Biol. Trace Elem. Res..

[67] Dumala N., Mangalampalli B., Chinde S., Kumari S.I., Mahoob M., Rahman M.F., Grover P. (2017). Genotoxicity study of nickel oxide nanoparticles in female Wistar rats after acute oral exposure. Mutagenesis.

[68] Reddy U.A., Prabhakar P.V., Mahboob M. (2017). Biomarkers of oxidative stress for in vivo assessment of toxicological effects of iron oxide nanoparticles. Saudi J. Biol. Sci..

[69] Kim D.E., Kim B., Shin H.S., Kwon H.J., Park E.S. (2014). The protective effect of hispidin against hydrogen peroxide-induced apoptosis in H9c2 cardiomyoblast cells through Akt/GSK-3β and ERK1/2 signaling pathway. Exp. Cell Res..

[70] Lai M.C., Liu W.Y., Liou S.S., Liu I.M. (2023). Hispidin in the Medicinal Fungus Protects Dopaminergic Neurons from JNK Activation-Regulated Mitochondrial-Dependent Apoptosis in an MPP+-Induced In Vitro Model of Parkinson’s Disease. Nutrients.

